# Asymmetric dynamics of edge exchange spin waves in honeycomb nanoribbons with zigzag and bearded edge boundaries

**DOI:** 10.1038/s41598-019-42742-5

**Published:** 2019-04-18

**Authors:** D. Ghader, A. Khater

**Affiliations:** 1 College of Engineering and Technology, American University of the Middle East, Egaila, Kuwait; 2 Department of Theoretical Physics, Institute of Physics, Jan Dlugosz University in Czestochowa, Am. Armii Krajowej 13/15, Czestochowa, Poland; 3 Department of Physics, University du Maine, 72085 Le Mans, France

**Keywords:** Magnetic properties and materials, Topological insulators, Two-dimensional materials

## Abstract

We report on the theoretical prediction of asymmetric edge spin waves, propagating in opposite directions at the boundaries of antiferromagnetic honeycomb nanoribbons with zigzag and bearded edges. The simultaneous propagation of edge spin waves along the same direction on both edges of the nanoribbons is forbidden. These asymmetric exchange spin waves at the edge boundaries are analogous to the nonreciprocal surface spin waves reported in magnetic thin films. Their existence is related to the nontrivial symmetry underlying these nanoribbons types. The discretized bulk and the edge exchange spin waves are calculated for the long wavelength part of the nanoribbon Brillouin zone (BZ), using the classical field spin wave theory and notably appropriate boundary conditions. In the absence of an external magnetic field in our study, the asymmetric edge spin waves propagate with equal frequencies and along opposite directions. The edge spin waves are characterized by linear dispersion relations for magnetically isotropic nanoribbons. For magnetically anisotropic nanoribbons, our calculations show that the energy gap between the edge and bulk spin waves is enhanced for both types of zigzag and bearded nanoribbons. The large energy gap separates the edge modes from overlapping the bulk ones. Also, we explain why our results for anisotropic zigzag nanoribbons go beyond previous studies based on a quantum approach in the linear spin wave approximation.

## Introduction

The continuum classical field theory, integrated with appropriate boundary conditions, plays a central role in the study of boundary magnetic excitations in thin films and layered nanostructures^1–22^. The allowed propagating and evanescent surface spin waves are generally determined by solving the bulk equations of motion consistently with the boundary condition equations. Classical field theory results for spin dynamics on thin and ultrathin magnetic films proved fundamental effects on both bulk and surface spin wave modes induced by the film’s symmetry, magnetic order, anisotropy, thickness and surface structure^23–27^. One fascinating fact in the field of thin film magnetism is the existence of nonreciprocal localization of surface spin waves in magnetic films with special symmetry and magnetic ordering. In these magnetic films, surface spin waves confined to opposite surfaces of the film travel in opposite directions, and are characterized by different frequencies in the presence of an external magnetic field. These films do not allow the simultaneous propagation of surface spin waves in the same direction on both boundaries. A comprehensive discussion on the symmetries giving rise to nonreciprocal surface spin waves in magnetic films can be found in^9^.

The important recent discovery of magnetism in two-dimensional (2D) and quasi-2D-layered van der Waals materials attracted substantial interest in their magnetic properties and excitations^28–54^. These materials present various types of magnetic order^29–32,34,35,42–44,49–51^ and are expected to open new opportunities in magnonics and spintronics. Spin waves have been recently detected in a variety of 2D and quasi-2D magnetic materials^31,34,41,48–51^, revealing novel characteristics for these magnetic excitations in consistency with some theoretical predictions.

Bounded 2D or quasi-2D magnetic materials present bulk and edge spin waves, in analogy with bulk and surface spin waves in magnetic thin films. Edge spin waves on semi-infinite and nanoribbon honeycomb monolayers with zigzag and armchair boundaries have been theoretically studied using quantum spin wave approaches, combined with boundary conditions^28,45,46,52^. The edge spin waves are found to display interesting and unconventional characteristics, notably for isotropic antiferromagnetic monolayers with zigzag edge boundaries, where Dirac edge modes are predicted.

The classical field spin wave theory is known to be equivalent to the quantum spin wave approach limited to the linear spin wave approximation. Despite its broad success in the study of surface spin waves, the classical field theory has not been systematically developed for edge spin waves in 2D materials until our recent study of long wavelength exchange spin waves on 2D antiferromagnetic nanoribbons with armchair edge boundaries^54^. Our previous study highlighted the important consequences on bulk and edge exchange spin waves induced by the finite width of the nanoribbon and by its magnetic anisotropy. The importance of magnetic anisotropy was also emphasized in a recent experiment on spin waves in *CrI*_3_ 2D honeycomb ferromagnet^48^. Although magnetic anisotropy is considered as an important ingredient to preserve the local magnetic moments against thermal fluctuations^48,53,55^, its effect on edge magnetic excitations in bounded 2D materials did not yet receive the deserved theoretical attention.

In the present work, we further develop the classical spin waves approach and apply it to study the bulk and edge exchange spin waves in 2D antiferromagnetic nanoribbons with zigzag and bearded edge boundaries, in the long wavelength part of the BZ. Appropriate boundary condition equations are derived taking into account the finite width of the nanoribbons. Solving the bulk equations of motion consistently with the boundary conditions yields the long wavelength edge and discretized bulk exchange spin waves for both types of nanoribbons, considered to be with and without magnetic anisotropy. Unlike previous theoretical studies^28,45,46,52^ where the boundary conditions are derived and solved on one edge of the nanoribbon, the derived boundary equations in our approach are solved simultaneously for both edges.

The nontrivial symmetry for the two types of nanoribbons is analyzed in our study and found to have important consequences for the characteristics of the edge spin waves. We theoretically predict that nanoribbons with zigzag or bearded edges boundaries forbid the simultaneous propagation of edge spin waves in the same direction on opposite edges. The allowed edge spin waves hence propagate in opposite directions on the opposite edges of the nanoribbons, as with the nonreciprocal surface spin waves in magnetic thin films.

**Figure 1:**
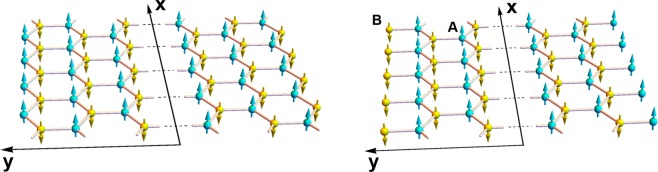
Schematic representations of a honeycomb nanoribbon with zigzag edge boundaries (left) and bearded edge boundaries (right).

The honeycomb nanoribbons with zigzag and bearded edge boundaries are presented in Fig. 1; they are considered infinite along the x-direction, and finite along the y-direction, with edges atx *x* = ±*d*. In terms of the honeycomb lattice constant *a*, the half-width *d* is respectively equal to $\frac{3n+2}{2\sqrt{3}}a$ and $\frac{3n+1}{2\sqrt{3}}a$ for zigzag and bearded edge nanoribbons (*n* is an integer number). In the Néel antiferromagnetic ordering state, the spins on A (blue) and B (yellow) sublattices are conventionally assumed aligned parallel and antiparallel to the z-axis. The left and right edge spins are of A and B types respectively in the zigzag edge nanoribbons. The opposite is assumed for the nanoribbons with bearded edge boundaries.

## Methods

We consider a semi-classical Heisenberg Hamiltonian with exchange interaction between the spins

$$ {\mathcal H} =J\,\sum _{\overrightarrow{r},\overrightarrow{\delta }}\,[{\overrightarrow{S}}_{\parallel }(\overrightarrow{r},t).{\overrightarrow{S}}_{\parallel }(\overrightarrow{r}+\overrightarrow{\delta },t)+\gamma {S}_{z}(\overrightarrow{r},t){S}_{z}(\overrightarrow{r}+\overrightarrow{\delta },t)]$$

where *t* is time, $\overrightarrow{r}=x\,\hat{x}+y\,\hat{y}$ is the position vector of a site on the honeycomb lattice, and $\overrightarrow{\delta }$ is the position vector of a nearest neighbor. *J* is the exchange constant and *γ* > 1 is an anisotropy parameter. The vector ${\overrightarrow{S}}_{\parallel }={S}_{x}\,\hat{x}+{S}_{y}\,\hat{y}$ represents the spin component in the plane of the honeycomb lattice.

Using the Bloch equations of motion for the magnetization ${\overrightarrow{M}}^{A}$ and ${\overrightarrow{M}}^{B}$ of the honeycomb sublattices^54^, the bulk wave equation describing the spin waves dynamics can be derived as

1 $$[\frac{1}{{\nu }^{2}}{\partial }_{t}^{2}-{\rm{\Delta }}+{\mu }^{2}]{ {\mathcal M} }^{A/B}(\overrightarrow{r},t)=0$$

with ∂_*t*_ = ∂/∂*t* and $${\rm{\Delta }}=\frac{{\partial }^{2}}{\partial {x}^{2}}+\frac{{\partial }^{2}}{\partial {y}^{2}}={\partial }_{x}^{2}+{\partial }_{y}^{2}$$.

The parameters in Eq. (1) are $\nu =\sqrt{\frac{3}{2}}\,\lambda \,JMa$ and $\mu =\sqrt{\frac{6({\gamma }^{2}-1)}{{a}^{2}}}$, with $M={M}_{z}^{A}=-\,{M}_{z}^{B}$ and *λ* is the gyromagnetic ratio. The complex variables ${ {\mathcal M} }^{A}$ and ${ {\mathcal M} }^{B}$ are defined in terms of the magnetization components as ${ {\mathcal M} }^{A}={M}_{x}^{A}+i{M}_{y}^{A}$ and ${ {\mathcal M} }^{B}={M}_{x}^{B}+i{M}_{y}^{B}$. Details on the derivation of Eq. (1) can be found in^54^.

Solving Eq. (1) for the bounded nanoribbon system, the variables ${ {\mathcal M} }^{A}$ and ${ {\mathcal M} }^{B}$ are written in the general form

2a $${ {\mathcal M} }^{A}={A}_{1}{e}^{i(\omega t-{k}_{x}x)}{e}^{qy}+{A}_{2}{e}^{i(\omega t-{k}_{x}x)}{e}^{-qy}$$

2b $${ {\mathcal M} }^{B}={B}_{1}{e}^{i(\omega t-{k}_{x}x)}{e}^{qy}+{B}_{2}{e}^{i(\omega t-{k}_{x}x)}{e}^{-qy}$$

Compared to previous studies^28,45,46,52^, we here adopt a more general form for the solutions, suitable for bounded systems where both *e*^±*qy*^ terms are physical. Here, *k*_*x*_ is the continuous wave vector along the infinite x-direction. The real and imaginary values of *q* correspond respectively to *evanescent* (edge), and *propagating* (bulk), spin waves in the y-direction along which the nanoribbon is finite.

Substituting equations (2) in the bulk wave Eq. (1) yields the dispersion relation

3 $$-\,{{\rm{\Omega }}}^{2}+\frac{3}{2}{a}^{2}({k}_{x}^{2}-{q}^{2})+9({\gamma }^{2}-1)=0$$

with the normalized frequency Ω defined as ${\rm{\Omega }}=\frac{\omega }{\lambda JM}$.

It is also possible to determine useful relations between the coefficients *A*_1_, *A*_2_, *B*_1_, and *B*_2_. With equations (2), the Bloch equations of motion for the magnetizations yield^54^

4a $$[(3\gamma -{\rm{\Omega }}){A}_{1}+\zeta \,{B}_{1}]{e}^{qy}+[(3\gamma -{\rm{\Omega }}){A}_{2}+\zeta \,{B}_{2}]{e}^{-qy}=0$$

4b $$[\zeta {A}_{1}+(3\gamma +{\rm{\Omega }}){B}_{1}]{e}^{qy}+[\zeta {A}_{2}+(3\gamma +{\rm{\Omega }}){B}_{2}]{e}^{-qy}=0$$

with $\zeta =3+\frac{{a}^{2}}{4}({q}^{2}-{k}_{x}^{2})$. Equations (4) hold for any *y* along the width of the nanoribbon. This is only possible if all coefficients of *e*^±*qy*^ are zeros. This yields the relations

5a $${A}_{1}=-\,\frac{3\gamma +{\rm{\Omega }}}{\zeta }{B}_{1}=-\,\frac{\zeta }{3\gamma -{\rm{\Omega }}}{B}_{1}$$

5b $${A}_{2}=-\,\frac{3\gamma +{\rm{\Omega }}}{\zeta }{B}_{2}=-\,\frac{\zeta }{3\gamma -{\rm{\Omega }}}{B}_{2}$$

which are consistent with the derived dispersion relation (2). We hence select {*A*_1_, *B*_2_} as the independent parameters and set ${B}_{1}=-\,\frac{3\gamma -{\rm{\Omega }}}{\zeta }{A}_{1}$ and ${A}_{2}=-\,\frac{3\gamma +{\rm{\Omega }}}{\zeta }{B}_{2}$.

The effective exchange fields for edge and bulk spins are different due to the reduced number of nearest neighbors for edge sites (one and two nearest neighbors for edge spins in nanoribbons with bearded and zigzag edges boundaries, respectively). Just like bulk fields, the edge exchange fields are derived from the Heisenberg Hamiltonian and the second order continuum expansion of the edge magnetizations^54^.

The exchange spin waves boundary conditions are derived from the requirement that edge spins satisfy the bulk equations of motion^8,9,12,15,17–22,54^ which yields the effective boundary equations

6 $${\overrightarrow{M}}_{e}^{A/B}\times ({\overrightarrow{H}}_{b}^{A/B}-{\overrightarrow{H}}_{e}^{A/B})=\overrightarrow{0}$$

where *e* and *b* stand for edge and bulk respectively. Nanoribbons with bearded or zigzag edges are characterized by opposite spins on the left and right edges. With only one type of spin on the edge boundaries, Eq. (6) yields a system of two simultaneous linear equations in the coefficients *A*_1_ and *B*_2_.

For nanoribbons with zigzag and bearded edges, the two boundary equations yield the matrix equations as follows:

$$\begin{array}{rcl}{M}_{1}(\begin{array}{c}{A}_{1}\\ {B}_{2}\end{array}) & = & (\begin{array}{cc}{{\rm{e}}}^{dq}{a}_{-} & {{\rm{e}}}^{-dq}{b}_{+}\\ {{\rm{e}}}^{-dq}{b}_{-} & {{\rm{e}}}^{dq}{a}_{+}\end{array})(\begin{array}{c}{A}_{1}\\ {B}_{2}\end{array})=0\\ {a}_{\pm } & = & (-\,\frac{3\gamma \pm \Omega }{\zeta })(1+\frac{aq}{\sqrt{3}}+\frac{{a}^{2}{q}^{2}}{6})+\gamma ,\\ {b}_{\pm } & = & (1-\frac{aq}{\sqrt{3}}+\frac{{a}^{2}{q}^{2}}{6})+(-\,\frac{3\gamma \pm \Omega }{\zeta })\gamma .\end{array}$$

for the zigzag edges, and

$$\begin{array}{rcl}{M}_{2}(\begin{array}{c}{A}_{1}\\ {B}_{2}\end{array}) & = & (\begin{array}{cc}{{\rm{e}}}^{dq}{c}_{-} & {{\rm{e}}}^{-dq}{d}_{+}\\ {{\rm{e}}}^{-dq}{d}_{-} & {{\rm{e}}}^{dq}{c}_{+}\end{array})(\begin{array}{c}{A}_{1}\\ {B}_{2}\end{array})=0\\ {c}_{\pm } & = & 2-\frac{{a}^{2}{k}_{x}^{2}}{4}+\frac{aq}{\sqrt{3}}+\frac{{a}^{2}{q}^{2}}{12}-\frac{2(3\gamma \pm \Omega )}{\zeta }\gamma ,\\  &  & {d}_{\pm }=(-\frac{3\gamma \pm \Omega }{t})(2-\frac{{a}^{2}{k}_{x}^{2}}{4}-\frac{aq}{\sqrt{3}}+\frac{{a}^{2}{q}^{2}}{12})+2\gamma \end{array}$$

for the bearded edges.

The determinant of the *M*_1_ and *M*_2_ matrices should vanish as a necessary condition for the existence of non-zero solutions. To ensure consistency in the developed theory, the determinant is calculated keeping only linear and quadratic terms in *k*_*x*_ and *q*, appropriate for long wavelength spin waves.

For the nanoribbons with zigzag edge boundaries, the determinant of *M*_1_ yields the characteristic boundary equation

7 $${f}_{1}=4\sqrt{3}aq(-1+{\gamma }^{2})+\{6(-1+{\gamma }^{2})+{a}^{2}[{k}_{x}^{2}+{q}^{2}(-5+2{\gamma }^{2})]\}\,tanh(2dq)=0$$

For the nanoribbons with bearded edge boundaries, the determinant of *M*_2_ yields a different characteristic boundary equation of the form

8 $${f}_{2}=4\sqrt{3}aq\,(\,-\,1+{\gamma }^{2})+\{12(\,-\,1+{\gamma }^{2})+{a}^{2}[{k}_{x}^{2}(5-3{\gamma }^{2})+{q}^{2}(\,-\,4+{\gamma }^{2})]\}\,tanh(2dq)=0$$

Equations (7) and (8) determine uniquely the boundary conditions. In turn the real solutions for these boundary equations determine the decay factors for edge exchange spin wave modes for respectively the nanoribbons with zigzag and bearded edge boundaries. Similarly, the imaginary solutions determine the allowed wavevectors for bulk modes in these nanoribbons. For the imaginary solutions, it is useful to substitute *q* = *ik*_*y*_ (*k*_*y*_ is the wavevector component along the y-direction for propagating modes) in Eqs (7) and (8). This yields the equivalent equations

9 $${g}_{1}=4\sqrt{3}a{k}_{y}({\gamma }^{2}-1)+\{6({\gamma }^{2}-1)+{a}^{2}[{k}_{x}^{2}+{k}_{y}^{2}(5-2{\gamma }^{2})]\}\,tan(2d{k}_{y})=0$$

for zigzag edged nanoribbons, and

10 $${g}_{2}=4\sqrt{3}a{k}_{y}({\gamma }^{2}-1)+\{12({\gamma }^{2}-1)+{a}^{2}[{k}_{x}^{2}(5-3{\gamma }^{2})-{k}_{y}^{2}(-4+{\gamma }^{2})]\}\,tan(2d{k}_{y})=0$$

for nanoribbons with bearded edge boundaries. Equations (9) and (10) do not admit continuous solutions for finite *d*. The allowed wavevector component *k*_*y*_ along the width 2*d* of the nanoribbon is hence discretized, and the number of solutions depends on this width.

## Results

### Isotropic nanoribbons

For magnetically isotropic nanoribbons (*γ* = 1), the boundary Eqs (9) and (10) for propagating spin waves reduce to the simple forms $({k}_{x}^{2}+3{k}_{y}^{2})tan(2d{k}_{y})=0$ and $(2{k}_{x}^{2}+3{k}_{y}^{2})tan(2d{k}_{y})=0$, respectively, or simply $tan(2d{k}_{y})=0$ for both nanoribbon edge types. The discrete solutions are handy for this case, given by ${k}_{y}=\pm \,n\pi \,/\,2d$ for both types, where *n* is a positive integer. The allowed values of *k*_*y*_ are nevertheless different for the two nanoribbon types, since the *d* values for the nanoribbon half width are structurally different for zigzag and bearded edge nanoribbons. This difference becomes particularly relevant in thin nanoribbons at small *d* values.

In an interval $[{k}_{y}=-\,{\rm{{\rm K}}},{k}_{y}=+\,{\rm{{\rm K}}}]$ of the long wavelength part of the Brillouin zone, the number of propagating exchange spin waves is hence given by the integer part of $\frac{2{\rm{{\rm K}}}d}{\pi }+1$. Here, we assume that + and −solutions of *k*_*y*_ belong to the same mode, which follows from equations (2). For edge modes, the boundary Eqs (7) and (8) reduce in the isotropic case to $({k}_{x}^{2}-3{q}^{2})tanh(2dq)=0$, and $(2{k}_{x}^{2}-3{q}^{2})tanh(2dq)=0$, respectively.

This yields edge exchange spin waves with linear factors $q=\pm \,\frac{1}{\sqrt{3}}{k}_{x}$ and $q=\pm \,\sqrt{\frac{2}{3}}{k}_{x}$ in nanoribbons with zigzag and bearded edge boundaries, respectively. The factors in this case are independent of the nanoribbons width and have larger absolute values in nanoribbons with bearded edge boundaries. Substituting in the normalized energy dispersion relation (3), yields the linear dispersion relations ${\rm{\Omega }}=|{k}_{x}|a$ and ${\rm{\Omega }}=\frac{1}{\sqrt{2}}|{k}_{x}|a$ for edge modes in nanoribbons with zigzag and bearded edges, respectively.

The calculation of the eigenvectors of *M*_1_ and *M*_2_ yield the amplitudes *A*_1_, *A*_2_, *B*_1_, and *B*_2_ for the determined edge and propagating solutions. For the edge modes, the eigenvectors for both matrices are (1, 0) and (0, 1). This is fundamentally different from the (1, 1) and (−1, 1) eigenvectors obtained in our previous study for edge spin waves on honeycomb nanoribbons with armchair boundaries^54^. Consequently, unlike nanoribbons with armchair edges, nanoribbons with zigzag and bearded edges do not allow edge spin waves propagating with the same *k*_*x*_ (or same direction) on both edges simultaneously. This is a direct consequence of the nontrivial symmetry underlying these nanoribbons.

**Figure 2:**
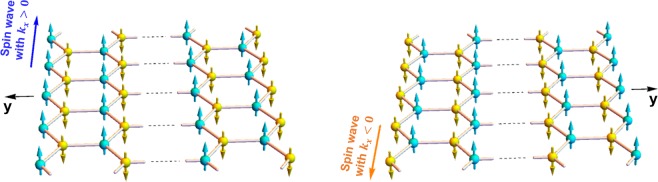
Schematic representations illustrating the complex symmetry underlying honeycomb nanoribbons with zigzag edge boundaries (see text for details).

Nanoribbons with bearded or zigzag edge boundaries are characterized by opposite spins on their two edges, which is different from the armchair edge nanoribbons where both sublattices are present on the boundaries. For armchair nanoribbons, the inversion of the coordinate axis along the finite width constitutes a symmetry operation leaving the system invariant. This is not the case for nanoribbons with bearded and zigzag edge boundaries characterized by a more complex symmetry operation. As illustrated in Fig. 2, the symmetry operation returning the nanoribbon with zigzag edges boundaries to an equivalent state constitutes of y-axis inversion followed by a 180° rotation about the y-axis. After the 180° rotation about the y-axis, an edge spin wave propagating with *k*_*x*_ > 0 at *y* = +*d* is transformed to an edge spin wave propagating with −*k*_*x*_ < 0 at *y* = −*d*. Edge spin waves in zigzag nanoribbons hence propagate in opposite directions on the two edges. The same argument holds for bearded nanoribbons.

For numerical applications at present, we select the half-width *d* as $\frac{71}{2\sqrt{3}}\approx 20.5$ and $\frac{70}{2\sqrt{3}}\approx 20.21$ for nanoribbons with zigzag and bearded edge boundaries, respectively; the lattice constant *a* is set to 1. The eigenvectors determine the spatial variation of the amplitudes of the edge spin wave across the finite width of the nanoribbon.

**Figure 3:**
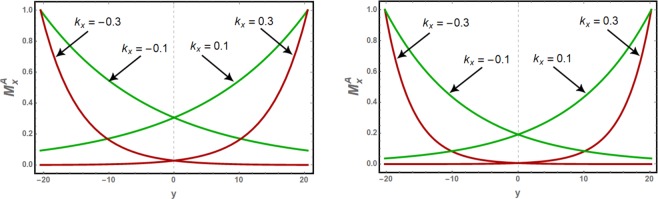
The spatial variation of the normalized amplitude ${M}_{x}^{A}$ of the asymmetric edge spin waves along the width of the isotropic nanoribbon for zigzag (*left*) and bearded (*right*) edge boundaries. The *d* half widths are respectively $71/(2\surd 3)\approx 20.50$ and $70/(2\surd 3)\approx 20.21$ for the respective nanoribbon types. The curves are plotted for selected values of *k*_*x*_.

In Fig. 3, the normalized ${M}_{x}^{A}$ amplitudes for edge spin waves are plotted along the width of the nanoribbons for *k*_*x*_ = 0.1 and 0.3. The normalized ${M}_{x}^{B}$ amplitudes are out of phase with respect to ${M}_{x}^{A}$ amplitude, following equation (5), and are not presented here. Compared to zigzag edged nanoribbons, evanescent modes in the nanoribbon with bearded edge boundaries are observed to decay faster into the bulk due to their larger decay factor.

The normalized energies for bulk and edge exchange spin wave modes can be calculated using the dispersion relation presented in Eq. (3). In the long wavelength part |*k*_*y*_| = ≤0.3 of the BZ, the boundary condition equation $tan(2d{k}_{y})=0$ yields four propagating modes for each of the nanoribbons with zigzag and bearded edge boundaries. The discrete wavevectors are |*k*_*y*_| = 0, $\frac{\sqrt{3}\,\pi }{71}$, $\frac{2\sqrt{3}\,\pi }{71}$, and $\frac{3\sqrt{3}\,\pi }{71}$ for the zigzag nanoribbon; these are slightly different for the bearded nanoribbon, namely |*k*_*y*_| = 0, $\frac{\sqrt{3}\,\pi }{70}$, $\frac{2\sqrt{3}\,\pi }{70}$, and $\frac{3\sqrt{3}\,\pi }{70}$.

**Figure 4:**
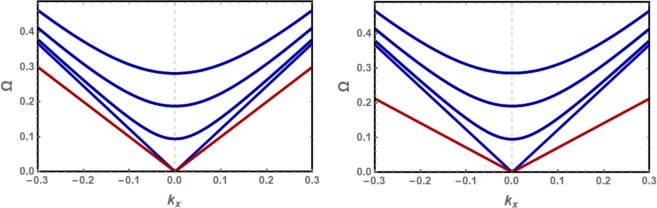
Normalized energy dispersion curves as a function of the continuous wavevector *k*_*x*_ for the discretized bulk modes (blue) and edge mode (red) for the isotropic nanoribbons with zigzag (*left*) and bearded (*right*) edges.

**Figure 5:**
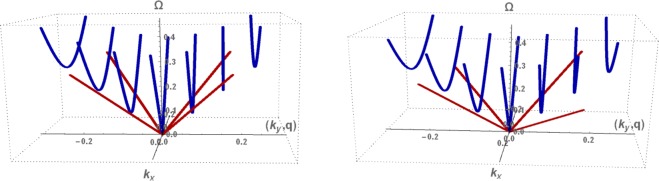
Normalized energy dispersion curves for long wavelength bulk (blue) and evanescent (red) exchange spin waves on a magnetically isotropic nanoribbon with zigzag edge boundaries (left) and bearded edge boundaries (right). The half-widths *d* are $71/(2\sqrt{3})$ and $70/(2\sqrt{3})$ for nanoribbons with zigzag and bearded edge boundaries, respectively. The curves are plotted as functions of the continuous *k*_*x*_ wavevector for the allowed (*k*_*y*_, *q*) values, *k*_*y*_ for (*bulk*) and *q* for (*evanescent*) modes.

In Fig. 4, the normalized energy dispersion curves for propagating (blue) and evanescent (red) modes are plotted as a function of the continuous wavevector *k*_*x*_ in the long wavelength part the honeycomb BZ. The same dispersion curves are plotted in Fig. 5 as functions of the continuous *k*_*x*_ wavevector for the allowed (*k*_*y*_, *q*) values, *k*_*y*_ for (*bulk*) and *q* for (*evanescent*) modes. The bulk dispersion curves are duplicated at positive and negative values of *k*_*y*_ following the infinite system conventional presentation of the dispersion curves.

As seen in Fig. 4, the bulk discrete modes are effectively the same in both nanoribbon types, and the discretization reduces the Dirac cone of the infinite system to a single linear dispersion curve for the bounded nanoribbons. Another effect of this discretization is the significant energy gaps induced between the allowed bulk spin waves. Compared to our previous results on armchair nanoribbons of comparable width^54^, the number of allowed bulk wavevectors is reduced which allows larger energy gaps between the bulk modes.

The edge modes are Dirac-like with energies below those of the propagating bulk modes, separated from them by an energy gap for |*k*_*x*_| > 0. In general they are characterized by a smaller group velocity. The energy gap between edge and propagating modes is larger for bearded than zigzag nanoribbons. For the zigzag nanoribbon, our results for the edge mode in the isotropic case are identical to previous studies^28,45^ based on the Holstein-Primakov quantum approach limited to the linear spin wave approximation.

### Anisotropy effects

**Figure 6:**
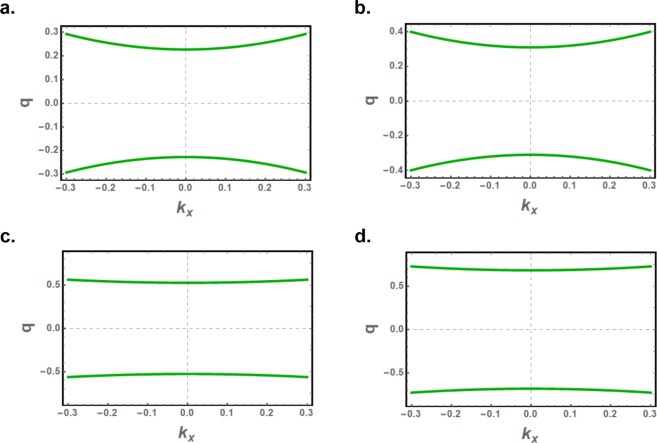
The *q* factors as a function of the continuous wavevector *k*_*x*_ for zigzag edged (**a**,**c**) and bearded edged (**b**,**d**) nanoribbons, with magnetic anisotropies *γ* = 1.01 (**a**,**b**) and *γ* = 1.04 (**c**,**d**). The nanoribbon half-width *d* is $71/(2\sqrt{3})$ and $70/(2\sqrt{3})$ for nanoribbons with zigzag and bearded edge boundaries, respectively.

We investigate the effect of nanoribbon magnetic anisotropy, with model values *γ* = 1.01 and *γ* = 1.04, on the bulk and edge exchange spin waves. The nanoribbon half-widths *d* are kept as before, namely as $71/(2\sqrt{3})$ and $70/(2\sqrt{3})$ for the two types of nanoribbons. The solutions of the decay factors *q*(*k*_*x*_) for edge modes are determined from the contour plot of Eqs. (7 and 8) and plotted in Fig. (6) as functions of *k*_*x*_.

**Figure 7:**
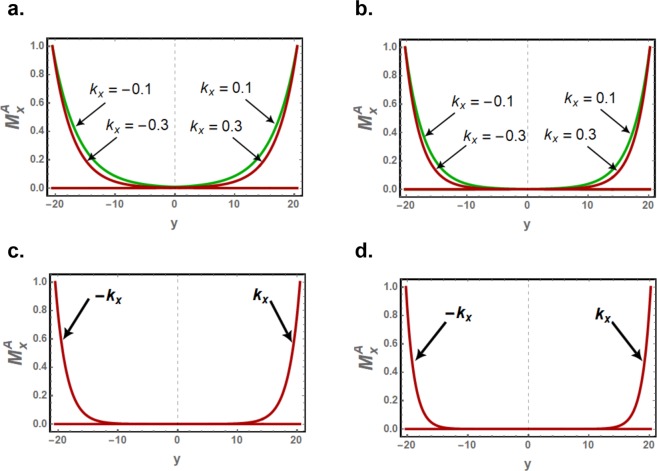
The spatial variation of the normalized amplitude ${M}_{x}^{A}$ of the asymmetric edge spin waves along the width of zigzag edged (**a**,**c**) and bearded edged (**b**,**d**) nanoribbons, with magnetic anisotropies *γ* = 1.01 (**a**,**b**) and *γ* = 1.04 (**c**,**d**). For *γ* = 1.04 case, the spatial variation is effectively independent of *k*_*x*_ in the long wavelength part of the Brillouin zone.

Slight anisotropies can significantly increase the decay factors which are then no more in linear dependence on *k*_*x*_. For *γ* = 1.04, for example, the decay factors are strikingly less dispersive as shown in Figs. (6c,6d), and the spatial variation of the edge spin wave amplitudes along the nanoribbon width becomes effectively independent of *k*_*x*_ in the long wavelength part. The spatial variations of the edge spin wave amplitudes are plotted in Fig. (7) for *γ* = 1.01 and *γ* = 1.04. Owing to these effects the penetration length for the edge spin waves is reduced significantly and the evanescent modes are increasingly confined to the edges.

For the two choices above of the anisotropy parameter, the number of bulk modes in the interval |*k*_*y*_| ≤ 0.3 is four for both nanoribbon types. The allowed wavevectors for the two types are very close in the relatively wide nanoribbons under study.

For the zigzag edge nanoribbon, the allowed *k*_*y*_ wavevectors are 0, ≈±0.075, ≈±0.151, and ≈±0.227 for *γ* = 1.01, and 0, ≈±0.075, ≈±0.149, and ≈±0.225 for *γ* = 1.04. As the anisotropy increases, the allowed wavevectors shift slightly toward the origin but the significant spacing between consecutive wavevectors is barely affected. We note that this shift of *k*_*y*_ towards the origin in zigzag edge nanoribbons with large anisotropy can allow additional propagating modes in the |*k*_*y*_| < 0.3 interval. For example, the number of modes in the zigzag nanoribbon with *γ* = 1.1 becomes five, with an additional mode at *k*_*y*_ ≈ ±0.299.

For the bearded edge nanoribbon, the allowed *k*_*y*_ wavevectors are 0, ≈±0.0769, ≈±0.1537, and ≈±0.2312 for *γ* = 1.01, and 0, ≈±0.0766, ≈±0.1533, and ≈±0.2301 for *γ* = 1.04. These allowed wavevectors also shift toward the origin though by slightly smaller values than those for the zigzag edge nanoribbon.

**Figure 8:**
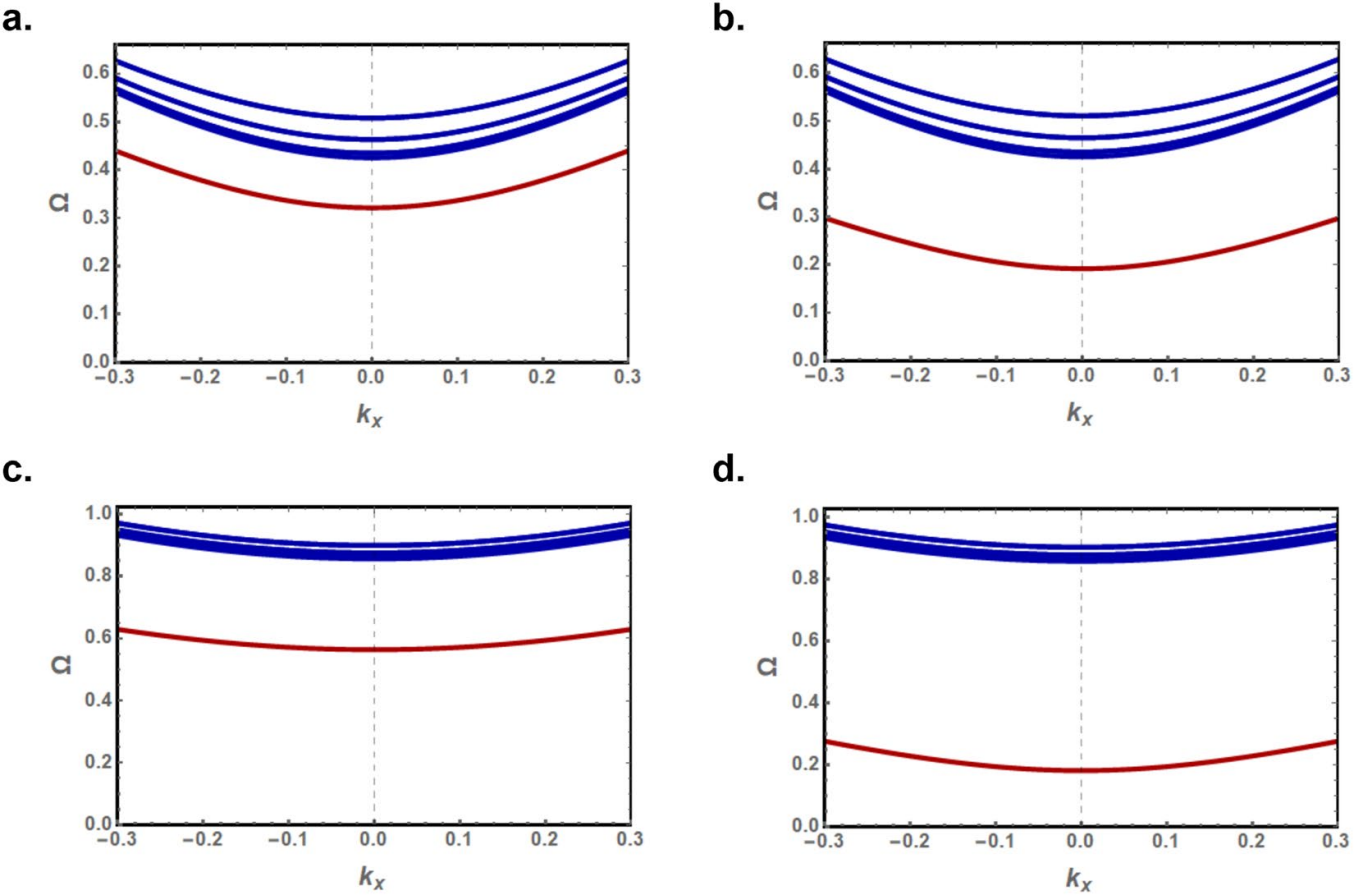
Normalized energy dispersion curves as a function of the continuous wavevector *k*_*x*_ for the long wavelength discretized bulk modes (blue) and edge mode (red) for the zigzag edged (**a**,**c**) and bearded edged (**b**,**d**) nanoribbons, with magnetic anisotropies *γ* = 1.01 (**a**,**b**) and *γ* = 1.04 (**c,d**).

**Figure 9:**
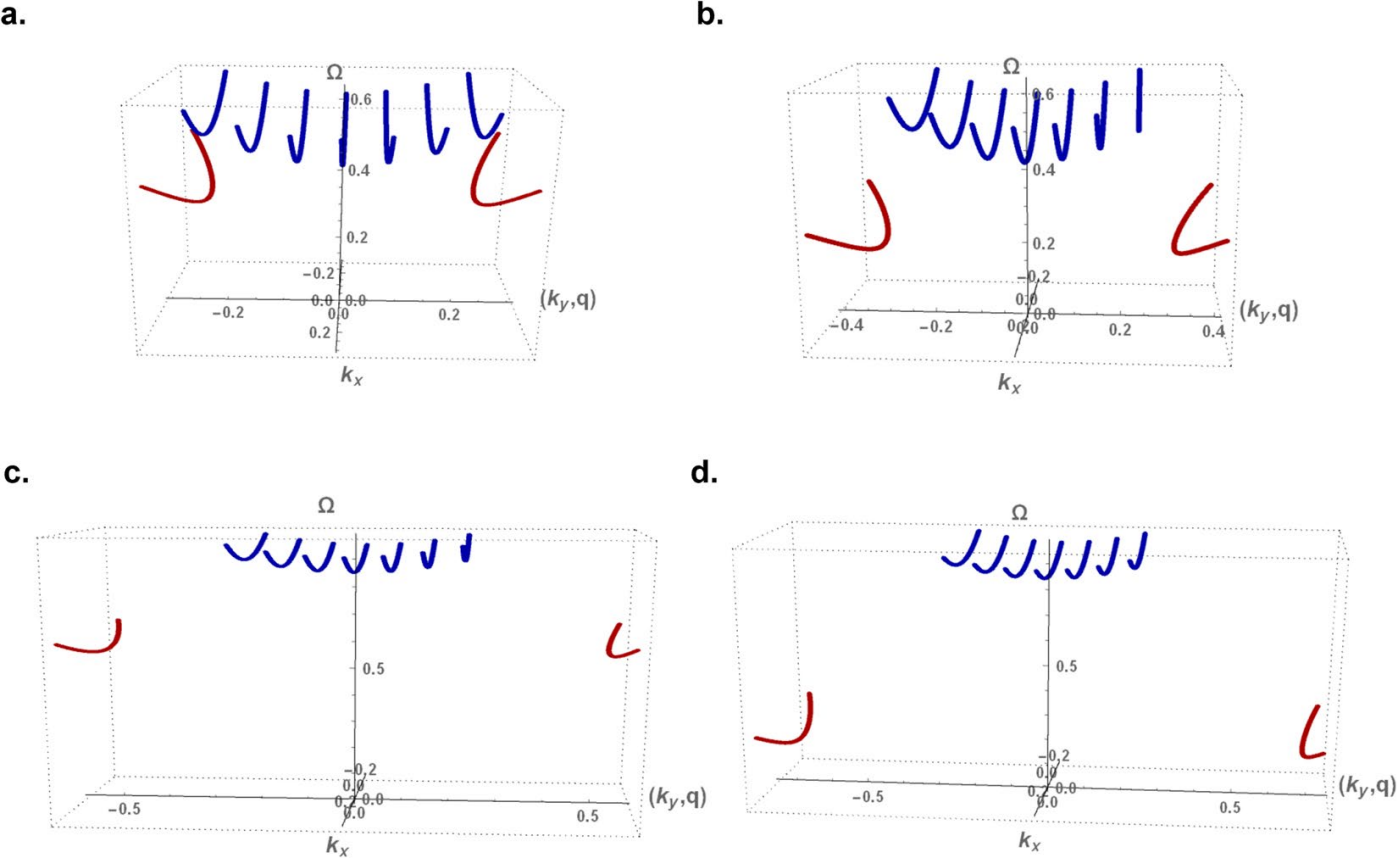
Normalized energy dispersion curves for long wavelength bulk (blue) and evanescent (red) exchange spin waves on the zigzag edged (**a**,**c**) and bearded edged (**b**,**d**) nanoribbons, with magnetic anisotropies *γ* = 1.01 (**a**,**b**) and *γ* = 1.04 (**c**,**d**). The curves are plotted as functions of the continuous *k*_*x*_ wavevector for the allowed (*k*_*y*_, *q*) values, *k*_*y*_ for (*bulk*) and *q* for (*evanescent*) modes.

The normalized energy dispersion curves for propagating (blue) and evanescent (red) modes are plotted as a function of the continuous wavevector *k*_*x*_ in Fig. (8) for *γ* = 1.01 and 1.04. The same dispersion curves are plotted in Fig. (9) as functions of the *k*_*x*_ wavevector for the allowed (*k*_*y*_, *q*) values, *k*_*y*_ for bulk and *q* for evanescent modes.

The normalized energies for the discrete bulk spin waves are effectively the same for the zigzag and bearded edge nanoribbons. Although the spacing between consecutive discrete *k*_*y*_ solutions are preserved, the energy gap between the bulk modes energies is reduced significantly with increasing anisotropy. Moreover, the dispersion of the bulk and edge mode energies decreases with anisotropy and the spin waves are characterized by slower group velocities. The density of states, however, is significantly enhanced.

A large energy gap is induced by anisotropy between the energies of propagating and edge spin waves in both nanoribbons. The gap is observed to increase with anisotropy. This large gap allows the separate excitation of long wavelength bulk and edge exchange spin waves. The edge mode energy is significantly lower in the nanoribbon with bearded edges boundaries due to the larger decay factor. An interesting observation is that the edge mode energy in this nanoribbon is lower in the *γ* = 1.04 case compared to the *γ* = 1.01.

## Discussion

A field theory approach is developed to calculate the long wavelength exchange spin wave modes on honeycomb nanoribbons with zigzag and bearded edge boundaries. Appropriate boundary conditions, which take into account the finite width of the nanoribbon, are derived from the requirement that the edge spins satisfy the bulk Bloch equations of motion. The boundary equations on both edges are solved simultaneously to determine the characteristic decay factors and the corresponding allowed discrete wave vector components for the edge and bulk modes respectively. Solving the bulk equations of motion in conjunction with the edge boundary condition equations, determines consequently the edge and bulk spin wave modes.

Our results for anisotropic nanoribbons with zigzag edge boundaries differ significantly from previous studies based on the quantum approach for two main reasons. First, we have used a more general framework for the magnetization dynamics. Second, the derived boundary equations based on the present formalism are solved simultaneously on both edges. This is particularly important for nanoribbon systems with narrow finite nanometric widths.

The bulk modes of the nanoribbon systems are found to be discretized due to the finite nanoribbon width, where it is shown characteristically that the Dirac cone in the infinite honeycomb lattice is reduced to a single Dirac mode. In the absence of magnetic anisotropy, the dispersion relation for edge modes is linear for both nanoribbons. The energy gap between the edge and propagating modes is found to be larger in bearded nanoribbons than in zigzag nanoribbons. The bearded edge spin waves are hence more spatially confined towards the edges compared to their confinement in zigzag edge spin waves.

Our theoretical study predicts that nanoribbons with zigzag and bearded edge boundaries forbid the simultaneous propagation of edge spin waves in the same direction on the opposite nanoribbon edges; they hence propagate in opposite directions on the two edges in analogy with the nonreciprocal surface spin waves in magnetic thin films. Similar to nonreciprocal surface spin waves, the asymmetric edge spin waves are expected to be very interesting for potential applications in magnonics. The existence of these edge spin waves is directly related to the nontrivial symmetry underlying the nanoribbons. Such edge modes, for example, cannot exist in nanoribbons with armchair boundaries. Despite their opposite propagation directions, the edge modes have the same propagation frequency in the absence of an external magnetic field, as neglected in the present study.

The subject of tunable magnetic anisotropy in 2D magnetic materials for technological requirements have already received significant attention^53–59^. The present work further promotes this research direction, as the magnetic isotropy is demonstrated to induce technologically desired effects on the spin waves spectrum in 2D antiferromagnetic nanoribbons. In particular, the nanoribbon magnetic anisotropy is found to significantly increase the energy gap between the edge and the bulk propagating modes for both zigzag and bearded nanoribbon types. For relatively high anisotropy, *γ* = 1.04, this gap is sufficient to separate energetically the edge modes from overlapping the bulk ones. Moreover, the asymmetric dynamics of edge spin waves is found to be robust against the magnetic anisotropy.

Edge spin wave excitations in 2D Dirac materials is a relatively recent field compared to surface spin waves which hence makes theoretical studies indispensable to support or even guide experimental work on these exotic materials. In this context, further development of the classical field theory beyond the scope of the present work, restricted to exchange and anisotropy interactions, is necessary.
